# Exploration of Bacterial Re-Growth as In Vitro Phenomenon Affecting Methods for Analysis of the Antimicrobial Activity of Chimeric Bacteriophage Endolysins

**DOI:** 10.3390/microorganisms10020445

**Published:** 2022-02-15

**Authors:** Ursula Kaspar, Nina Schleimer, Evgeny A. Idelevich, Sonja Molinaro, Karsten Becker

**Affiliations:** 1 Institute of Medical Microbiology, University Hospital Münster, 48149 Münster, Germany; ursula.kaspar@lzg.nrw.de (U.K.); ninaschleimer@web.de (N.S.); evgeny.Idelevich@med.uni-greifswald.de (E.A.I.); 2 Friedrich Loeffler-Institute of Medical Microbiology, University Medicine Greifswald, 17475 Greifswald, Germany; 3 Microcoat Biotechnologie GmbH, 82347 Bernried, Germany

**Keywords:** endolysin, bacteriophage, *Staphylococcus aureus*, re-growth, in vitro stability, susceptibility testing, test standardization

## Abstract

Drug alternatives to combat methicillin-resistant *Staphylococcus aureus* (MRSA) in human and animal healthcare are urgently needed. Recently, the recombinant bacteriophage endolysins, PRF-119 and its successor substance HY-133, have proven to be highly active against various *S. aureus* clonal lineages and to exhibit a very rapid bactericidal effect when standard methods for susceptibility testing are applied. Along with subsequent growth curve experiments, a re-growth phenomenon was observed in vitro necessitating its clarification for the assessment of the agent’s stability and activity as well as for methodological aspects of endolysin testing in general. Distinct in vitro parameters were comparatively examined applying also scanning electron microscopy, fluorescence assays and SDS-PAGE analysis. The shape and material of the culture vessels as well as the shaking conditions were identified as factors influencing the in vitro stability and activity of HY-133. The highest function maintenance was observed in plain centrifuge tubes. Based on this, the conditions and parameters of assays for testing the antimicrobial activities of phage endolysins were determined and adjusted. In particular, shear forces should be kept to a minimum. Our results form the basis for both future test standardization and re-growth-independent experiments as prerequisites for exact determination of the antimicrobial activities of engineered endolysins.

## 1. Introduction

Methicillin-resistant *Staphylococcus aureus* (MRSA) is a major cause of community- and healthcare-associated human and animal infections worldwide [1]. As it is a serious threat for individual patients and additional socioeconomic burden for healthcare systems, infection control measures including de-colonization strategies may help to prevent infections caused by healthcare-, community- and livestock-associated MRSA [2,3,4,5]. Increasing resistance towards topical agents for nasal MRSA de-colonization including mupirocin and biocides necessitates the search for alternative MRSA-active topical antibacterials [6,7,8,9]. Phages and, in particular, bacteriophage-derived lysins have recently been considered as an alternative therapeutic strategy to combat also the escalating problem of resistance in human and animal healthcare [10]. HY-133 (HYPharm GmbH, Bernried, Germany) is a chimeric bacteriophage endolysin, which may open novel strategies for nasal de-colonization of patients carrying *S. aureus*, in particular MRSA. Subsequent to the determination of the specific *S. aureus* activity of the predecessor agent PRF-119 [11], HY-133’s activity against different clonal lineages and phenotypic variants of the *S. aureus* complex including livestock-associated MRSA, *Staphylococcus schweitzeri* strains, and different clinical and laboratory strains exhibiting the small-colony variant (SCV) phenotype was verified by means of standard methods for testing antimicrobial activities [12,13,14]. In a recent in-depth study, time–kill curves revealed a very rapid bactericidal effect of HY-133 even with low concentrations [15]. Unexpectedly, re-growth was observed after prolonged incubation times, the mechanisms of which remained largely unclear [15]. This phenomenon was also observed in experiments with graft surface-adherent cells where HY-133 showed moderate bactericidal effect [16]. Here, we aimed to elucidate the nature of this re-growth phenomenon and to study in detail the in vitro test conditions that favor or prevent it.

## 2. Materials and Methods

### 2.1. Minimum Inhibitory Concentration (MIC) and Minimum Bactericidal Concentration (MBC) Determination

Minimum inhibitory concentration (MIC) of HY-133 against *S. aureus* strain ATCC 29213 was determined in sterile 96-well microplates (Greiner Bio One International, Kremsmünster, Austria) following the Clinical and Laboratory Standards Institute (CLSI) guidelines, as previously described [12,17,18]. Briefly, two-fold dilution series of HY-133 were prepared in cation-adjusted Mueller-Hinton broth (CAMHB, Becton Dickinson, Franklin Lakes, NJ, USA), with final concentrations ranging from 0.016–8 µg/mL. *S. aureus* ATCC 29213 was inoculated via direct colony suspension from stationary phase cultures grown overnight on Columbia blood agar (Becton Dickinson). A final concentration of 5 × 10^5^ CFU/mL was used for inoculation of the 96-well microplates. 

For determination of the minimum bactericidal concentration (MBC), 10 µL aliquots of culture from each microwell displaying the MIC and concentrations above were inoculated onto tryptic soy agar (TSA, Becton Dickinson) plates and incubated at 37 °C overnight. The MBC was defined as the concentration of HY-133 resulting in <500 CFU/mL (killing rate of 99.9%) [18]. Tests were performed in triplicate, and median values were taken for analysis. 

### 2.2. Time–Kill Assays

Killing kinetics of HY-133 against *S. aureus* strain ATCC 29213 under different conditions were analyzed by means of time–kill curves following CLSI guideline M26-A [19]. In brief, cultures were grown on Columbia blood agar plates (Becton Dickinson) at 37 °C overnight. Several colonies were then inoculated in a pre-culture (10 mL tryptic soy broth, TSB, Becton Dickinson) and cultivated at 37 °C and 160 rpm. After 3 h, pre-cultures were adjusted in CAMHB (Becton Dickinson) to starting inocula of 5 × 10^5^ CFU/mL. Time–kill kinetics were evaluated under different conditions as follows:

(i) Evaluation of bactericidal concentrations of HY-133 against *S. aureus* ATCC 29213 over time was based on MIC values determined via broth microdilution methodology. Time–kill curves were carried out in glass baffled flasks (GBF; Schott AG, Mainz, Germany). The 1-, 2-, 4-, 16-, 32-, and 64-fold MIC of HY-133 (HYPharm GmbH, Bernried, Germany) was analyzed.
(ii) Interval dosage experiments were carried out adding 16-fold MIC of HY-133 to the ATCC 29213 culture at time point 0 h and after (i) 2 h, (ii) 1 h and 3 h, (iii) 2 h and 4 h, or (iv) 1 h, 2 h, and 3 h, respectively. Experiments were performed in GBFs (Schott AG).
(iii) The maintenance of HY-133 activity against *S. aureus* ATCC 29213 in time–kill curve experiments (CLSI guideline M26-A) [19] was determined by pre-incubation of the substance (16-fold MIC dissolved in CAMHB (Becton Dickinson)) for different time spans (0.25 h, 0.5 h, 1 h, and 2 h) under given conditions (GBFs, 37 °C, 160 rpm) before adding the starting inoculum of *S. aureus* ATCC 29213. GBFs (Schott AG) were further used for time–kill curves.
(iv) The influence of different shearing forces, culture flask materials, and their forms was evaluated by applying the 16-fold MIC of HY-133 against *S. aureus* strain ATCC 29213 in different culture vessels (Table 1). Incubation was carried out shaking at 160 rpm.

For all experiments, viability counts were carried out at 0 h, 1 h, 2 h, 4 h, 6 h, 8 h, 24 h, and 48 h of incubation via plating culture aliquot dilutions on TSA (Becton Dickinson) in triplicates. After incubation at 37 °C overnight, colonies were counted. Growth and sterile controls were included in each experiment and all experiments were performed at least in triplicate.

### 2.3. Scanning Electron Microscopy

To visualize the impact of different culture flask materials on HY-133’s efficacy and structure, samples were prepared in the same way as described for time–kill assays in different culture vessels. Briefly, the 16-fold MIC of HY-133 was applied against *S. aureus* strain ATCC 29213 in (i) GBFs, (ii) PBFs, and (iii) CTs. Small pieces (0.5 × 0.5 cm) of the respective materials were added into the corresponding culture flasks during incubation. Cultures were kept for 3 h at 37 °C and 160 rpm. Subsequently, the small pieces of the different materials were removed from cultures and prepared for scanning electron microscopy (SEM) as previously described [20,21]. In brief, samples were fixed in 2.5% (*v*/*v*) glutaraldehyde in 0.1 M phosphate buffered saline (PBS) (pH 7.5) overnight followed by three times washing with PBS [22]. Samples were then post-fixed in 1% (*w*/*v*) osmium tetroxide in 0.1 M PBS (pH 7.5) and washed again in PBS (pH 7.5) for 3–5 min. Removal of water was accomplished by a graded water-ethanol series of 10–15 min steps (1 × 30%, 1 × 50%, 2 × 70%, 1 × 90%, and 2 × 100%). For high-resolution field-emission scanning electron microscopy (FESEM), samples underwent critical point drying using liquid CO_2_. Samples were attached to an aluminum carrier, then coated with 20 nm gold via sputter coating. Images were taken with an S-800 SEM (Hitachi Ltd., Tokyo, Japan) at room temperature and 20 kV acceleration voltage.

### 2.4. Fluorescence Assays

HY-133 protein activity under different conditions was determined using the HY-133 Activity Assay Kit (version 3.0, HYPharm GmbH) following the manufacturer’s instructions. For this, HY-133 was suspended in PBS at a final concentration of 400 µg/mL. The suspension was aliquoted into GBFs, PBFs, CTs, and BCTs and incubated at 37 °C either with shaking (160 rpm) or stationary (0 rpm). Samples were drawn after 6 h, 24 h, and 48 h and used for activity measurements via fluorescence of a target substrate. The substrate is an oligopeptide containing the fluorescence dye N-methyl-anthraniloyl (N-Me-Abz) and a quencher (2,4-dinitrophenol, DNP) inhibiting the fluorescence signal when the peptide is intact. These components are linked with amino acids in the following order: DNP–L-Lys–(Gly)5–D-Ala–L-Lys–N-Me-Abz. This structure corresponds to the pentaglycine interpeptide bridge characteristic for the *S. aureus* peptidoglycan layer [23,24]. The active HY-133 cleaves the substrate at the cross bridge between Gly and D-Ala, leading to the fluorescence of the substrate via loss of the quencher function. The fluorescence signal was observed under stimulation and emission wavelengths of 340/30 nm and 440/40 nm, respectively (Synergy HTX Multi-Mode Microplate Reader, Biotek, Winooski, VT, USA). The specific activity (U/mg) was calculated by means of a calibration curve generated with a standard provided by the manufacturer (HYPharm GmbH). A positive and a negative control, provided in the kit, were included in each measurement. The experimental procedure was carried out in three biological replicates. 

### 2.5. Sodium Dodecyl Sulfate Polyacrylamide Gel Electrophoresis (SDS-PAGE)

HY-133 stability was evaluated via sodium dodecyl sulfate polyacrylamide gel electrophoresis (SDS-PAGE). Samples were drawn and cleaned up with the ReadyPrepTM 2-D Cleanup Kit (Bio-Rad, Hercules, CA, USA) following the manufacturer’s instructions. Samples were resuspended in 1× Laemmli sample buffer, incubated at 98 °C for 10 min, and then loaded on an 8–16% Mini-ProteanR TGX Stain-FreeTM Gel (Bio-Rad) at a final quantity of 45 µg each. Gels were stained in Coomassie brilliant blue overnight. Protein bands were visualized in the ChemiDoc Touch Imaging System (Bio-Rad).

## 3. Results

**Figure 1:**
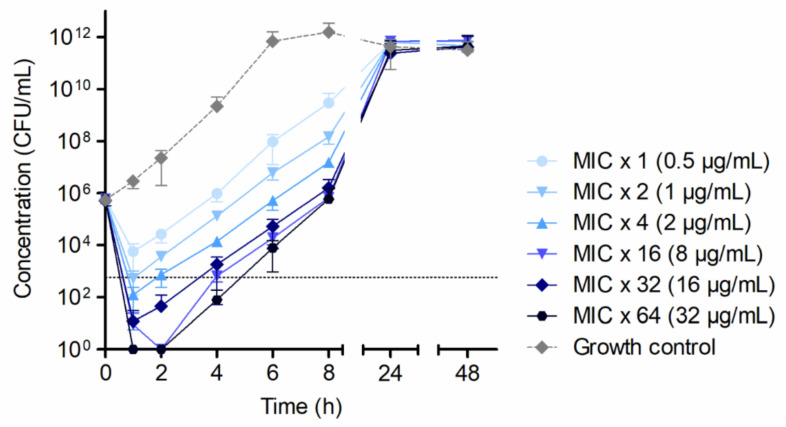
Time–kill curves applying different HY-133 concentrations against *S. aureus* strain ATCC 29213 showing killing and subsequent bacterial re-growth. Plot shows mean values for log_10_ of the numbers of CFU/mL versus time. The threshold (dotted line) implicates the ≥3−log_10_ decrease in CFU/mL. Time–kill curves were performed in triplicate (mean ± standard deviation). A culture of *S. aureus* strain ATCC 29213 without HY-133 was always included as a growth control (dashed line).

By microdilution, median MIC and MBC of each 0.5 µg/mL of HY-133 were determined for *S. aureus* ATCC 29213. Based on microdilution data, 1-, 2-, 4-, 16-, 32-, and 64-fold MICs of HY-133 were applied in time–kill experiments. The killing kinetics showed a decrease in CFU/mL within the first hour of the experiment for all concentrations of HY-133 applied. A minimum of the four-fold MIC induced a >3−log_10_ reduction in CFU/mL in the first hour of the experiment, a minimum of the 16-fold MIC led to a decrease below the detection limit of <100 CFU/mL (Figure 1). The application of higher concentrations (32- and 64-fold MIC, respectively) yielded similar killing kinetics as the 16-fold MIC. After prolonged incubation, an increase in CFU/mL was observed for all concentrations of HY-133 applied, resulting in cell numbers mirroring those of the growth control culture after 24 h (Figure 1).

**Figure 2:**
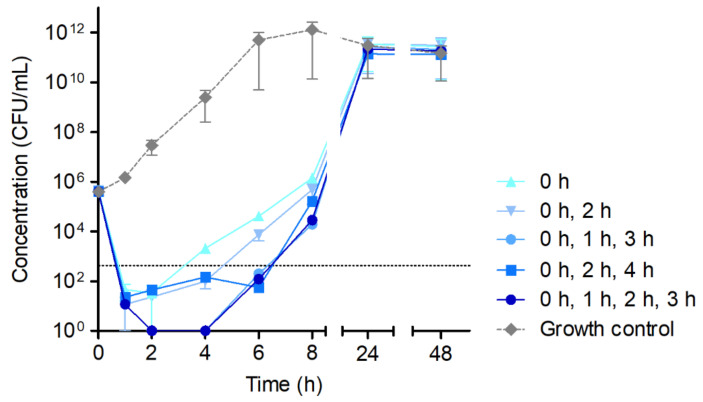
Time–kill curves of the 16-fold MIC of HY-133 applied at multiple time points against *S. aureus* strain ATCC 29213. Plot shows mean values for log_10_ of the numbers of CFU/mL versus time. The threshold (dotted line) implicates the ≥3−log_10_ decrease in CFU/mL. Time–kill curves were performed in triplicate (mean ± standard deviation). A culture of *S. aureus* strain ATCC 29213 without HY-133 was always included as a growth control (dashed line). The times given in the legend show the time points of HY-133 application.

In order to estimate a possible compensatory effect of the multiple application of HY-133, time–kill curves were carried out under repeated addition of the 16-fold MIC of HY-133 at different time points (Figure 2). Here, HY-133 was applied at time points (i) 0 h, (ii) 0 h and 2 h, (iii) 0 h, 1 h, and 3 h, (iv) 0 h, 2 h, and 4 h, and (v) 0 h, 1 h, 2 h, and 3 h, respectively. An increase in CFU/mL was observed for all approaches with a slightly slower re-growth of all cultures treated with HY-133 at multiple time points compared to the culture treated with a single dosage of HY-133 at time point 0 h. After 24 h, CFU/mL of all test vessels were equal to those of the growth control culture (Figure 2). 

**Figure 3:**
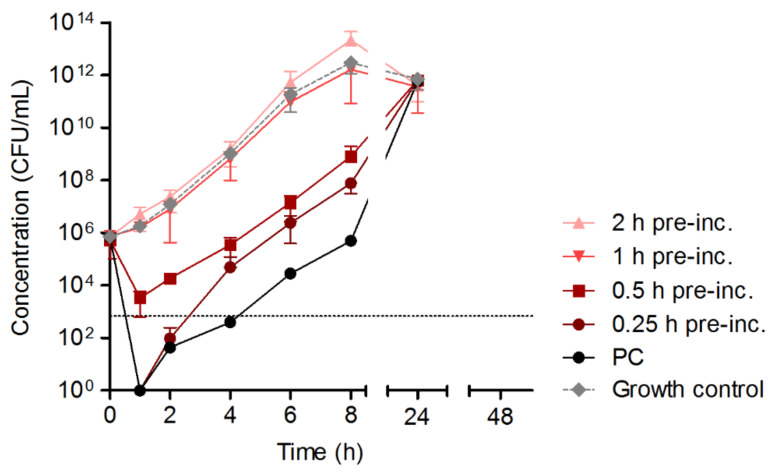
Time–kill curves of the 16-fold MIC of HY-133 applied against *S. aureus* strain ATCC 29213 subsequent to bacteria-free HY-133 pre-incubation (exposure times of 0.25 h to 2 h). Plot shows mean values for log_10_ of the numbers of CFU/mL versus time. The threshold (dotted line) implicates the ≥3−log_10_ decrease in CFU/mL. Time–kill curves were performed in triplicate (mean ± standard deviation). Cultures of *S. aureus* strain ATCC 29213 (i) with freshly applied HY-133 and (ii) without HY-133 were included as positive and growth control, respectively. PC, positive control (fresh HY-133); pre-inc., pre-incubation of 16-fold MIC of HY-133 in CAMHB.

A potential loss of the in vitro activity of HY-133 over time was further analyzed via pre-incubation of the substance under the particular conditions of a time–kill experiment as previously established. For this, HY-133 was incubated for different time intervals in GBFs shaking at 160 rpm and tempered at 37 °C before *S. aureus* ATCC 29213 culture was added to the medium. HY-133’s pre-incubation for 0.5 h led to a reduction in cell numbers <3 log_10_. Longer pre-incubation times of 1 h or 2 h, respectively, led to a complete loss of the enzyme’s function, shown in growth curves similar to the curve of the growth control culture (Figure 3).

**Figure 4:**
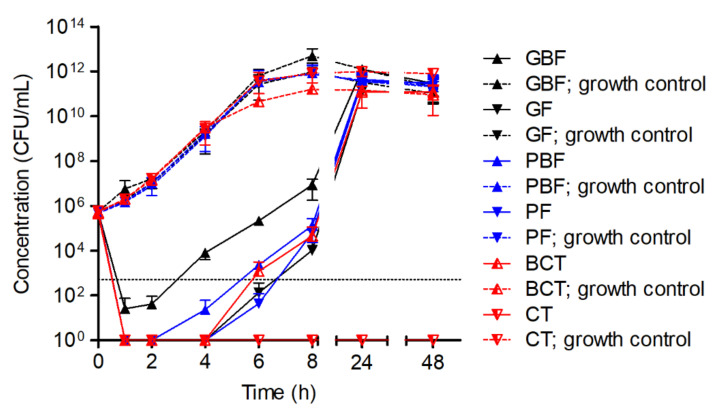
Time–kill curves of the 16-fold MIC of HY-133 against *S. aureus* strain ATCC 29213 incubated in different culture vessel types shaking at 160 rpm. Plot shows mean values for log_10_ of the numbers of CFU/mL versus time. The threshold (dotted line) implicates the ≥3−log_10_ decrease in CFU/mL. Time–kill curves were performed in triplicate (mean ± standard deviation). A culture of *S. aureus* strain ATCC 29213 without HY-133 was always included as a growth control (dashed lines) for each vessel type. GBF, glass baffled flask; GF, glass flask; PBF, plastic baffled flask; PF, plastic flask; BCT, baffled centrifuge tubes; CT, centrifuge tube.

The causality of the environmental conditions in the time–kill curve experimental setting and the in vitro efficacy of HY-133 was further explored by analyzing the impact of the culture flask’s form and material. For this, GBFs, GFs, PBFs, PFs, BCTs, and CTs (see Table 1) were utilized. For incubation at 160 rpm, the earliest re-growth of *S. aureus* strain ATCC 29213 was observed for GBFs, whereas cultures in GFs, PBFs, and PFs remained under the detection threshold (<100 CFU/mL) for a longer period of time. The most efficient killing of *S. aureus* cells was shown for the cultures in CTs. Here, no re-growth of the culture was observed over the experiment’s time span of 48 h (Figure 4).

**Figure 5:**
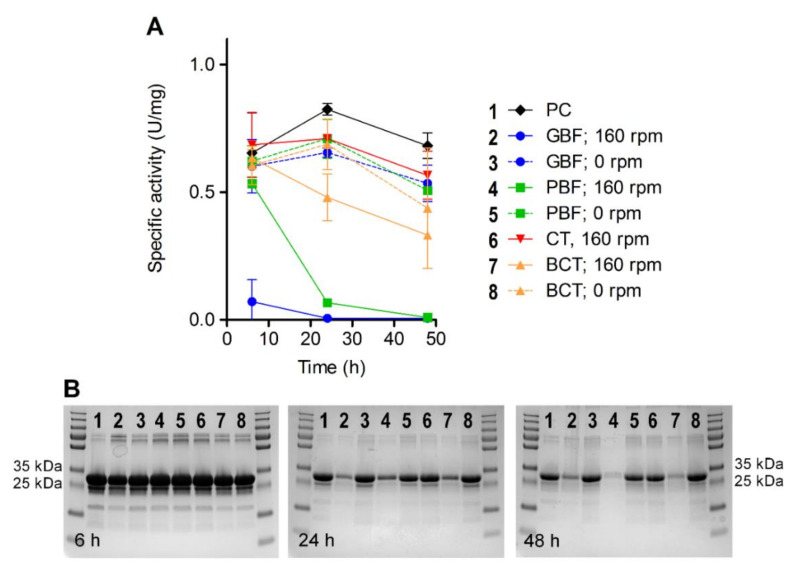
Measurements of HY-133’s specific activity (**A**) and stability (**B**) upon incubation under different in vitro conditions. Measurements were carried out after 6 h, 24 h, and 48 h of incubation. As a positive control, HY-133 was freshly applied at each measurement. PC, positive control (fresh HY-133); GBF, glass baffled flask; PBF, plastic baffled flask; CT, centrifuge tube; BCT, baffled centrifuge tube.

The impact of the flask material and shearing force on the stability and function of HY-133 was confirmed by analyzing the enzyme’s specific activity under different conditions over time. For both baffled culture flasks, a strong reduction of HY-133’s activity was observed after 24 h at 160 rpm. In the same culture flasks, the activity remained stable for 24 h, when incubation was carried out without shaking. The highest function maintenance for HY-133 over time was observed in plain CTs, where after 48 h the enzyme’s function was still comparable to fresh HY-133 (Figure 5A).

These results were further mirrored by SDS-PAGE analysis showing the stability of the protein over time. Here, for all approaches where HY-133 had been incubated shaking at 160 rpm and at 37 °C in baffled culture flasks, after 24 h, bands at expected height (32 kDa) became much weaker compared to those of the other approaches (Figure 5B). Bands on the SDS gel remained much stronger for the samples when HY-133 was incubated in the same culture flasks at 37 °C but without shaking. This difference was apparent irrespective of the culture flask material. In contrast to this, when incubated in plain CTs, the stability of the protein was maintained over 48 h even when the tubes were shaken at 160 rpm (Figure 5B).

**Figure 6:**
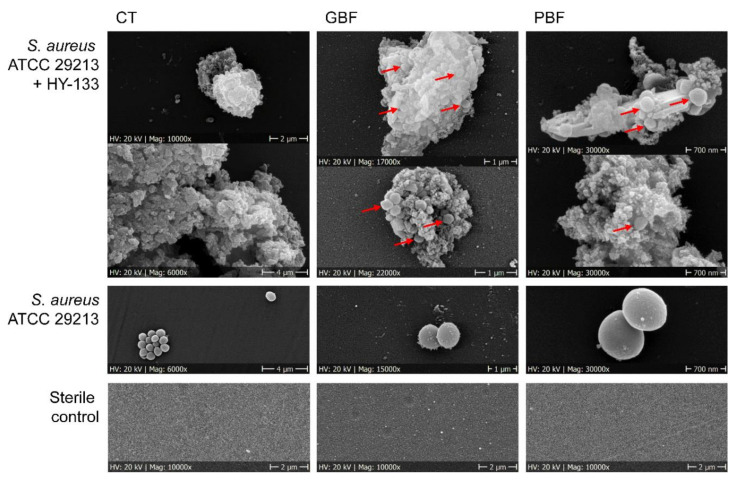
Scanning electron microscopic analysis of the impact of different culture flask materials on the in vitro stability and activity of HY-133 against S. aureus strain ATCC 29213. Red arrows mark *S. aureus* cells enclosed in agglomerates of inactive enzyme. Magnifications are provided below each picture. CT, centrifuge tube; GBF, glass baffled flask; PBF, plastic baffled flask.

SEM examinations of the activity of HY-133 on *S. aureus* cells upon incubation at 37 °C and 160 rpm revealed shrunk and irregular empty cell envelopes resembling *S. aureus* ghost cells (using GBFs and PBFs) (Figure 6). In CTs, no ghost cells were detected in HY-133-treated cultures.

## 4. Discussion

Today, we face the challenge of bringing new antimicrobials to the market. Optimal novel antimicrobial substances should bear the following characteristics: (i) be more refractory to resistance development; and (ii) be as specific as possible to a particular pathogen in order to protect the beneficial microbiota. Phage endolysins may address these requests and, thus, are promising candidates for alternative antibiotic substances. Their mode of action is independent of the metabolism of the bacteria, as they simply form holes in the bacterial cell wall through digestion of the peptidoglycan. Even if doses of endolysins were sub-lethal, a formation of resistance in the target bacteria would be unlikely, as the respective regions in the bacterial cell wall are highly conserved [25,26]. Furthermore, bacteriophage lysins tend to only aim at a certain bacterial species or subspecies [27]. Moreover, lysing bacterial cells within minutes, bacteriophage endolysins exhibit a very fast activity compared to classical antibiotics [15].

For the chimeric phage endolysins PRF-119 and the successor HY-133, rapid and specific action against *S. aureus*, mediated by the cell wall binding domain (i.e., lysostaphin), was already demonstrated in several studies [11,12,13,14,15]. For the precursor PRF-119, a “trailing effect” was described for microdilution assays, shown in point-like growth in wells with agent concentrations higher than the MIC [11]. Since this effect might have indicated a certain deficiency in stability of the protein structure, the stability-optimized HY-133 agent was designed showing no “trailing effect” in standard microdilution susceptibility assays. However, subsequent in-depth investigations of the in vitro activity of HY-133 in time-kill studies unexpectedly revealed re-growth of *S. aureus* strains, a phenomenon that had not occurred in previous microdilution susceptibility assays with HY-133 [11,12,13,14,15]. It is unlikely that the re-growth was caused by the development of resistance. In previous studies of our group, the survivor cells were susceptible to endolysin when sub-cultured and retested [15]. Thus, suboptimal standard in vitro conditions used for testing were concluded as a working hypothesis. Consequently, this study was intended to elucidate interfering assay conditions as a prerequisite for standardization of bacteriophage endolysin testing and as a methodological basis for future in vitro studies dealing with engineered bacteriophage products. 

To test its susceptibility, the test strain ATCC 29213 was first subjected to the broth microdilution assay. It was found that both MIC and MBC of HY-133 for ATCC 29213 were generally low (0.5 µg/mL) and comparable to MICs and MBCs against other *S. aureus* strains tested before [13,14]. The identical values for MIC and MBC further proved an absence of phenotypic antimicrobial tolerance in strain ATCC 29213 [19,28]. In time–kill experiments, re-growth of strain ATCC 29213 was demonstrated, as already observed in the context of previous studies [12,15]. A 64-fold increase of the MIC or multiple doses of HY-133 at different time points during the experiment could not compensate for this effect. Via systematic pre-incubation of HY-133, we found that the enzyme completely lost its action against the test strain after only 1 h of incubation under the previously used test conditions. Shape and material of the culture vessel hitherto used for the experimental procedure were identified as causes for this loss of efficacy. The combination of unfavorable form and material was shown to lead to a loss of function of the enzyme when shaken at 160 rpm. The shaking of the culture flask, however, is an important factor in in vitro testing for (i) an adequate distribution of both substance and bacteria and (ii) an adequate supply of oxygen in the culture in order to provide ideal growth conditions for aerobic bacteria. By using a round vessel with a soft plastic surface, we were able to show that shaking at 160 rpm lost its negative impact on HY-133’s in vitro activity completely.

The analysis on HY-133’s functional activity further substantiated this finding. Here, HY-133 induced a stable fluorescence signal of the test substrate over the time span of 48 h when incubated in plain CTs, comparable to the enzyme’s activity when freshly applied. This was also mirrored by demonstrating the stability of HY-133 applying SDS-PAGE analysis. Protein bands of HY-133 incubated in CTs at 160 rpm were comparable to those of the freshly tested endolysin. By contrast, baffled culture flasks led to a decrease in both activity and stability of HY-133 when incubated at the same speed. This decrease was observed for the different flask materials tested. However, the strongest effect was seen in GBFs, whereas the weakest effect was obvious in the BCTs. This is another hint that, apart from the culture flask form, also its material is decisive for the enzyme’s activity. Similar to its precursor PRF-119, HY-133 consists of a cysteine- and histidine-dependent amidohydrolase/peptidase (CHAP) domain from the endolysin of phage K acting as enzymatic active domain (EAD) and a lysostaphin-based cell wall-binding domain (CBD) [11,14,29,30,31]. The EAD and the CBD are connected through a linker peptide. Although already shortened and optimized compared to the linker in PRF-119, this part of the recombinant agent might be the weak part in in vitro tests applying relatively harsh environmental assay conditions for those linker peptide-containing proteins [11]. However, this does not necessarily imply instability of the agent in vivo.

SEM analysis underpinned the growth curve results. Applying different assay conditions, HY-133 activity resulted in the absence of intact *S. aureus* cells and formation of conglomerates consisting of the content of lysed bacterial cells and possible residues of inactive endolysin. These conglomerates also became macroscopically visible after a prolonged time span of 24 h (data not shown) and were mirrored by loss of protein bands in SDS-PAGE analysis. Besides these conglomerates, bacterial cell ghosts appear with empty irregular cell envelopes in PBFs and GBFs. These bacterial ghosts were detectable on the top as well as within the conglomerates. In contrast to this, in plain CTs, no cell ghosts were seen. Despite its pronounced in vitro susceptibility apparent in all culture flasks, it is probably that the highest proportion of active HY-133 could be maintained in the plain CTs leading to an efficient and complete cell wall destruction before formation of the conglomerates. Due to the ultra-fast mode of action of the endolysin, large amounts of the cell content of many *S. aureus* cells are simultaneously released and one could speculate that the resulting cell debris may enclose occasional a few *S. aureus* cells before being lysed. Thus, in vitro, HY-133 might be virtually “a victim of its own success” leading to protective effects which are not to be expected under in vivo conditions with lower bacterial cell count. Such a “protective effect by hiding” could be hypothesized in the case of PBFs and GBFs. This might also be the reason for the inefficiency of repeated doses of the endolysin at successive time points. In this case, the bacterial cells enclosed were not reached by the freshly added HY-133 anymore. The prolonged maintenance of HY-133 in plain CTs and the absence of ghost cells is also in line with the results of (i) the time-kill curves, (ii) the activity, and (iii) the stability assays. Accordingly, we highly recommend the utilization of plain CTs for killing kinetic analyses and other experiments in which high-speed shaking is necessary.

## 5. Conclusions

In summary, we were able to identify distinct factors impairing the in vitro stability and, hence, the in vitro activity of HY-133. By determining the influence of assay-related environmental conditions and material properties on the structure, stability and activity of this endolysin, we elucidated optimized conditions for in vitro testing of HY-133. In particular, shear forces should be kept as low as possible. If shaking is unavoidable, tubes with a plain surface should be used. These findings should be considered for future attempts to establish standard recommendations for testing and analyzing the in vitro activities of bacteriophage endolysins, in particular for those with chimeric structure. The observed protective effect on very few *S. aureus* cells, probably associated with extremely rapid “overkilling”, could explain the phenomenon of re-growth. Although most likely limited to in vitro conditions, this warrants further investigation and should be considered for future treatment strategies with bacteriophage endolysins.

## Figures and Tables

**Table 1:** Different culture vessels utilized for analysis of the impact of in vitro assay conditions on HY-133.

Culture Vessel	Material	Volume (mL)	Manufacturer
Glass baffled flask (GBF)	Glass	100	Schott AG, Mainz, Germany
Glass flask (GF)	Glass	100	Schott AG, Mainz, Germany
Plastic baffled flask (PBF)	Non-pyrogenic polycarbonate	125	Corning Incorporated, Corning, NY, USA
Plastic flask (PF)	Non-pyrogenic polycarbonate	125	Corning Incorporated, Corning, NY, USA
Baffled centrifuge tube (BCT) ^1^	Polypropylene	50	Greiner Bio One International, Kremsmünster, Austria
Centrifuge tube (CT)	Polypropylene	50	Greiner Bio One International, Kremsmünster, Austria

^1^ BCTs were produced by clamping x-formed pieces of polypropylene into the bottom of plain CTs under sterile conditions.

## Data Availability

Data are contained within the article.
